# SARS-CoV-2 and RSV external quality assessment on molecular detection and variant typing in European expert laboratories in 2025

**DOI:** 10.1128/jcm.00258-26

**Published:** 2026-09-17

**Authors:** Kim C. Heimsch, Kamelia R. Stanoeva, Ingmar Visser, Yannis Rahou, Tobias Bleicker, Alawiya Reslan, Danielle Perez-Bercoff, Eeva K. Broberg, Chantal B. E. M. Reusken, Victor M. Corman, Christian Drosten, Adam Meijer, Marie-Anne Rameix-Welti

**Affiliations:** 1 Institute of Virology, Charité-Universitätsmedizin Berlin, corporate member of Freie Universität Berlin and Humbolt-Universität zu Berlin 111617, Berlin, Germany; 2 Centre for Infectious Disease Control, National Institute for Public Health and the Environment (RIVM) 10206, Bilthoven, the Netherlands; 3 Molecular Mechanisms of Multiplication of Pneumoviruses Unit (M3P), Institut Pasteur, Université Paris-Saclay, Université de Versailles St. Quentin, Université Paris Cité, UMR 1173 (2I), INSERM, Centre National de Reference Virus des Infections Respiratoires (CNR VIR), Assistance Publique des Hôpitaux de Paris 27058, Paris, France; 4 European Centre for Disease Prevention and Control (ECDC), Stockholm, Sweden; 5 German Centre for Infection Research (DZIF), associated partner site Charité 459706, Berlin, Germany; 6 Labor Berlin-Charité Vivantes 646268 , Berlin, Germany; Wadsworth Center - NYSDOH, Albany, New York, USA

**Keywords:** molecular diagnostics, external quality assessment, Europe, RSV, SARS-CoV-2, COVID-19

## Abstract

We herein report on the fourth SARS-CoV-2 wet-lab external quality assessment (EQA) and the second organized by the AURORAE consortium to assess the diagnostic capabilities of European expert public health laboratories. Diagnostic capabilities were investigated regarding SARS-CoV-2 and, for the first time, respiratory syncytial virus (RSV) groups A and B. A 10-sample panel containing various SARS-CoV-2 variants, RSV lineages, a common cold coronavirus, and a negative control was produced and validated. Participants were invited by the European Centre for Disease Prevention and Control (ECDC) and asked to submit results and assay details via electronic forms. Thirty-seven laboratories from 32 European countries participated. All panel samples were identified correctly via molecular detection by 23 participating laboratories (23/37; 62%). Commercial assays were used most often (23/37; 62%), in comparison to in-house assays (14/37; 38%), or a combination of both (8/37; 22%). RSV groups were correctly determined by 20 laboratories (20/37; 54%). The optional task of SARS-CoV-2 variant typing was performed by 20 laboratories using next-generation sequencing. Thirteen laboratories successfully generated SARS-CoV-2 sequences. Six laboratories performed optional RSV sequencing, with only two obtaining sequences. The current and previous EQAs show that molecular detection of SARS-CoV-2 within Europe has improved over time and is now at a consistently high level. Molecular detection of RSV samples with low viral load was challenging, as was lineage identification for low-concentration specimens. Routine monitoring of diagnostic and surveillance assays via EQAs remains key to maintaining rapid and long-lasting public health laboratory response systems.

**IMPORTANCE:**

External quality assessments (EQAs) are essential for ensuring reliable and consistent diagnostic testing. They provide an objective way to evaluate laboratory performance, identify weaknesses, and drive improvements, as well as ensuring compliance with accreditation standards. Furthermore, participating in EQAs strengthens the contributions to public health disease surveillance and threat detection on national and transborder scales. During the SARS-CoV-2 pandemic, EQAs were vital for maintaining accuracy in the setting of increasing testing demands and emerging variants. Broadening of diagnostic testing to a wider panel of viruses, including respiratory syncytial virus (RSV), requires continuous performance monitoring to ensure accuracy and reliability. Ongoing development of vaccines and therapeutics, which may drive the emergence of new mutations, further underscores the need for regular evaluation of test effectiveness. By standardizing methods and reducing errors, EQAs ensure trustworthy and comparable results across laboratories. Regular participation in EQAs strengthens molecular diagnostics and variant surveillance.

## INTRODUCTION

External Quality Assessments (EQAs) play a crucial role in ensuring the accuracy and reliability of laboratory diagnostics. In particular, they help to assess the sensitivity and specificity of molecular assays and the accuracy of genomic sequencing, which is essential for variant surveillance and outbreak investigation. By participating in EQAs, laboratories contribute to global efforts in monitoring and controlling respiratory virus outbreaks through high-quality, reproducible diagnostics.

Previously, three SARS-CoV-2 wet-lab European EQAs were organized in 2020, 2021, and 2023 through collaborations between the European Centre for Disease Prevention and Control (ECDC) and contractors such as the AURORAE consortium (1–3). Over the time of these three EQAs, molecular detection of SARS-CoV-2 improved in European expert laboratories, especially for low-concentration samples. The percentage of laboratories correctly identifying all samples increased from 12% in 2020 to 75% in 2021, reaching 90% in 2023 (3).

Respiratory syncytial virus (RSV) was included in the 2025 wet-lab EQA described here due to its significant impact on health and the recent development of new prevention strategies. RSV is a leading cause of severe lower respiratory tract infections in infants and is increasingly recognized as a major health threat for older adults, resulting in substantial hospitalizations and deaths each year (4, 5). Given the recent approval of RSV vaccines for older adults and new effective monoclonal antibodies for infants (6), robust public health surveillance is essential to monitor the impact of these interventions at the clinical and virological levels. RSV is now a key target within European integrated sentinel surveillance systems for respiratory viruses, alongside influenza virus and SARS-CoV-2. In this context, it is important to integrate RSV into EQA schemes to enable laboratories to evaluate and ensure the accuracy and reliability of their RSV diagnostic tests.

## MATERIALS AND METHODS

### Participants

This EQA was implemented by the AURORAE consortium in coordination with the ECDC. Invitations were sent to the Operational Contact Points for Influenza (Microbiology), the Operational Contact Points for COVID-19 (Microbiology), the National Focal Points for Viral Respiratory Diseases, the Western Balkans and Türkiye Observer National Focal Points for Viral Respiratory Diseases, and the Western Balkans and Türkiye Influenza and COVID-19 (Microbiology) Contact Points for Operations. Therefore, the participating laboratories were mainly (national) reference laboratories. The list of participating laboratories is given in the Acknowledgements.

### Panel composition

The EQA panel consisted of 10 samples: four of SARS-CoV-2 (B.1.1.7, JN.1, and XEC variants), four of RSV group A (RSV-A A.D.5.1) or group B (RSV-B B.D.E.1), one control sample containing the human common cold coronavirus HCoV-229E, and one negative control with phosphate-buffered saline (PBS). The SARS-CoV-2 alpha variant was chosen as constant lineage with the previous SARS-CoV-2 wet-lab EQAs (1–3). A new batch of EQA samples was generated from the same viral stock, with slight variation in viral load. The JN.1 and XEC variants were chosen as lineages circulating at the time of the EQA. RSV-A A.D.5.1 (EPI_ISL_18568018) and RSV-B B.D.E.1 (EPI_ISL_18775428) were chosen because they were widely circulating lineages in mid-2024.

Two different concentrations of SARS-CoV-2 XEC, RSV-A, and RSV-B were chosen to evaluate assay sensitivity. The exact composition of the EQA panel is detailed in Table 1.

**TABLE 1:** EQA panel composition and performance of 37 laboratories participating in the European SARS-CoV-2 and RSV EQA, 2025

					Correct molecular detection		Correct RSV group A or B		Correct SARS-CoV-2 variant/RSV lineage determination	
Sample ID	Virus	Lineage	GISAID ID	Cps/ µL^*a*^^,*b*^	No./total labs	%	No./total labs with RSV positive	%	No./total labs performing NGS	%
1014	SARS-CoV-2	B.1.1.7	EPI_ISL_18796793	2.4E + 02	35/37	95	–	–	0/20	0
1023	SARS-CoV-2	JN.1	EPI_ISL_18373905	5.3E + 06	37/37	100	–	–	12/20	60
1032	SARS-CoV-2	XEC	EPI_ISL_19616050	6.2E + 03	37/37	100	–	–	5/20	25
1033	SARS-CoV-2	XEC	EPI_ISL_19616050	7.9E + 02	37/37	100	–	–	1/20	5
1038	RSV	A 2023-26978 (A.D.5.1)	EPI_ISL_18568018	3.1E + 04	36/37	97	30/36	81	2/7	29
1034	RSV	A 2023-26978 (A.D.5.1)	EPI_ISL_18568018	1.3E + 03	31/37	84	25/31	68	0/7	0
1041	RSV	B 2023-28159 (B.D.E.1)	EPI_ISL_18775428	1.3E + 04	35/37	95	31/35	84	2/7	29
1037	RSV	B 2023-28159 (B.D.E.1)	EPI_ISL_18775428	5.2E + 02	26/37	70	21/26	57	0/7	0
1016	HCoV^*c*^	229E	–	3.0E + 02	37/37	100	–	–	–	–
2010	PBS^*c*^^,*d*^	–	–	–	37/37	100	–	–	–	–

^*a*^ cps/µL, copies/µL.

^*b*^ For SARS-CoV-2-containing samples, an in-house assay targeting the E gene was used (7). Samples containing the common cold HCoV 229E were tested using a specific in-house RT-PCR assay (8). RSV-containing samples were tested using the RealStar RSV RT-PCR Kit 3.0 (Altona Diagnostics).

^*c*^ Correct molecular detection is “negative”.

^*d*^ PBS, phosphate-buffered saline.

### Panel validation, testing instructions, and panel dispatch

All SARS-CoV-2 samples were isolated from clinical specimens using Vero E6 cells or Vero E6 cells overexpressing TMPRSS2 and cultivated in Dulbecco’s modified Eagle medium (DMEM) supplemented with 2% fetal calf serum (FCS). HCoV-229E BN_1 (9) was stocked using HUH-7 cells cultured in DMEM supplemented with 2% FCS. All RSV samples were isolated and passaged once on HEp-2 cells (ATCC-CCL-23) maintained in Eagle’s minimum essential medium (MEM) supplemented with 10% FCS and 1% penicillin–streptomycin solution (5,000 U/mL). All cells were grown at 37°C in 5% CO_2_.

All EQA samples were inactivated by incubation with 0.05% β-propiolactone (BPL) at 4°C for 14–16 h, followed by incubation at 37°C for 2 h. To confirm successful inactivation of SARS-CoV-2, three consecutive passages were performed in Vero E6 cells cultured in DMEM supplemented with 2% FCS.

The inactivated samples were diluted with PBS to the desired concentration and lyophilized in screw-cap vials. At Charité-Universitätsmedizin Berlin, all SARS-CoV-2-containing samples were quantified using the E-gene target in an RT-PCR-based in-house assay (8). The HCoV-229E-containing sample was quantified using a specific RT-PCR-based in-house protocol (9). For quantification of the RSV-containing samples, the RealStar RSV RT-PCR Kit 3.0 (Altona Diagnostics) was used. Prior to the EQA trial, sample validation was performed independently at three laboratories: Charité-Universitätsmedizin Berlin, the National Institute for Public Health and the Environment of the Netherlands (RIVM), and the Institute Pasteur (IP) in Paris (Table S1). Regarding the optional sequencing exercise, two out of the three pre-testing laboratories could generate a sequence and correctly identify all panel samples.

### Data collection

Results were reported via an online survey form (hosted on the EU survey platform). The mandatory tasks were molecular detection of all samples, positive identification of all SARS-CoV-2- or RSV-containing samples, and RSV group identification (A or B) for the RSV-containing samples. Participants were asked to provide information about the extraction methods and types of RT-PCRs (in-house protocol and/or commercial kit) for each target and each sample, score their samples (positive, negative, and inconclusive), and provide information about cycle threshold (Ct) values. Voluntarily, participants could perform lineage determination via sequencing or typing PCR for all SARS-CoV-2- and RSV-containing samples. For this task, participants could report the determined lineage and/or SARS-CoV-2 signature mutations. Participants were asked to report on the sequencing methods and platforms used.

### Evaluation of results

Results of the molecular detection were scored as in previous EQAs (2, 3): correctly reported samples (presence or absence of SARS-CoV-2 or RSV RNA), 0 penalty points; inconclusive results, 1 penalty point; and false-negative or false-positive results, 2 penalty points. For RSV group determination, results were scored as correct A or B, RSV not determined, or RSV negative. Lineage determination of SARS-CoV-2 and RSV was scored as specific, unspecific, not determined/negative/failing, or false.

### Statistical analysis

Data cleaning and analysis were performed using Python/Jupyter Lab and R (version 4.5.0). Figures were prepared using GraphPad Prism (version 10.0) and ECDC Map Maker tool (EMMa). Statistical differences in Ct values from different assays were calculated using one-way ANOVA in GraphPad Prism (version 10.0).

## RESULTS

### Participation

**Fig 1:**
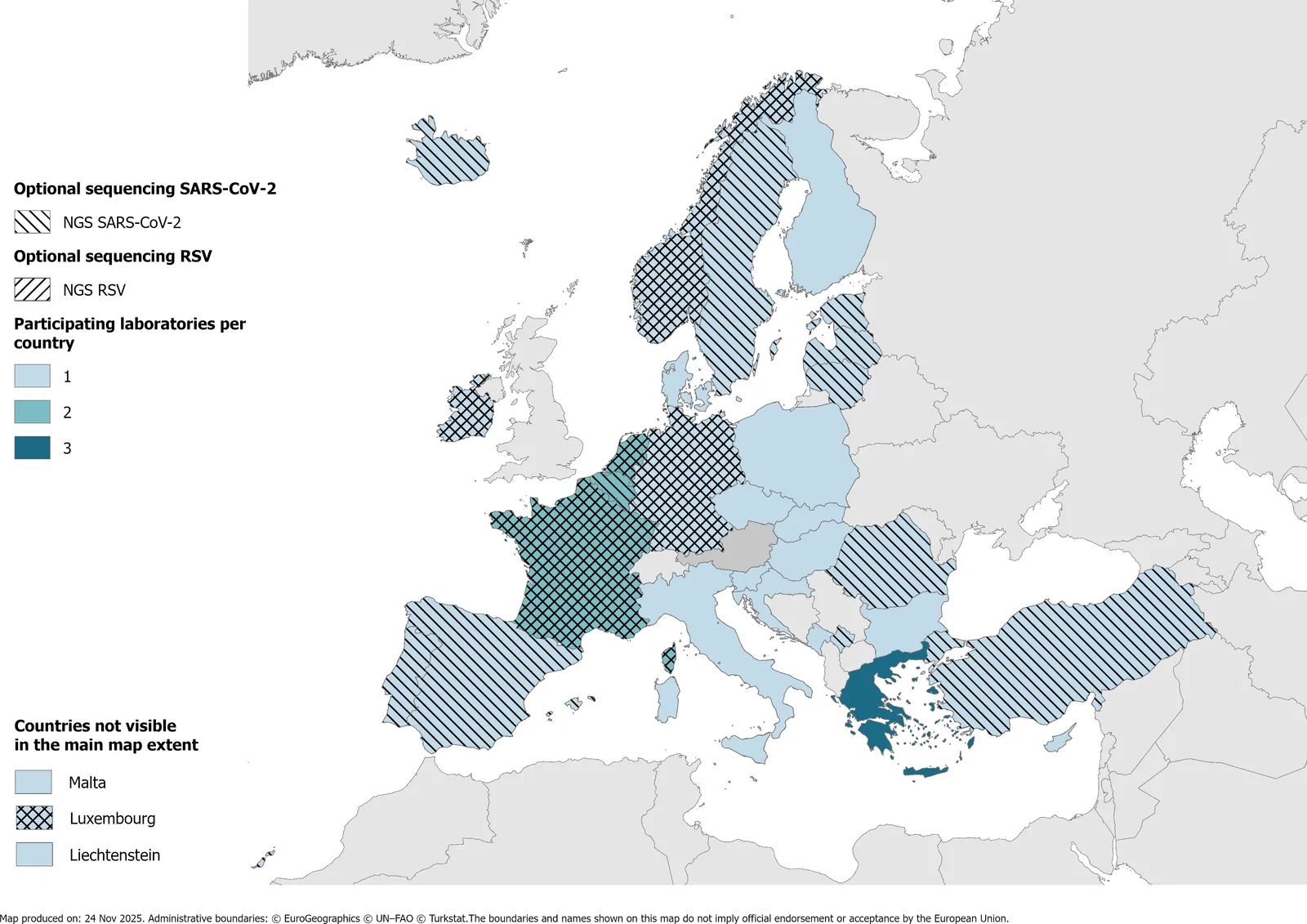
Map of participating laboratories. The number of participating laboratories per country is color-coded. Participation in the optional sequencing part is represented by diagonal lines. Map created using the ECDC Map Maker 181 tool (EMMa).

Thirty-seven expert laboratories from 32 countries participated in the wet-lab SARS-CoV-2/RSV EQA 2025 (Fig. 1), including 26 EU and three EEA (European Economic Area) countries, as well as three laboratories from the Western Balkans and Türkiye. In most cases, one laboratory per country participated (28/32 countries; 87.5%). For three countries (3/32; 9.4%), two laboratories participated, and in one country (1/32; 3.1%), three laboratories participated.

### Molecular detection of SARS-CoV-2 and RSV

**Fig 2:**
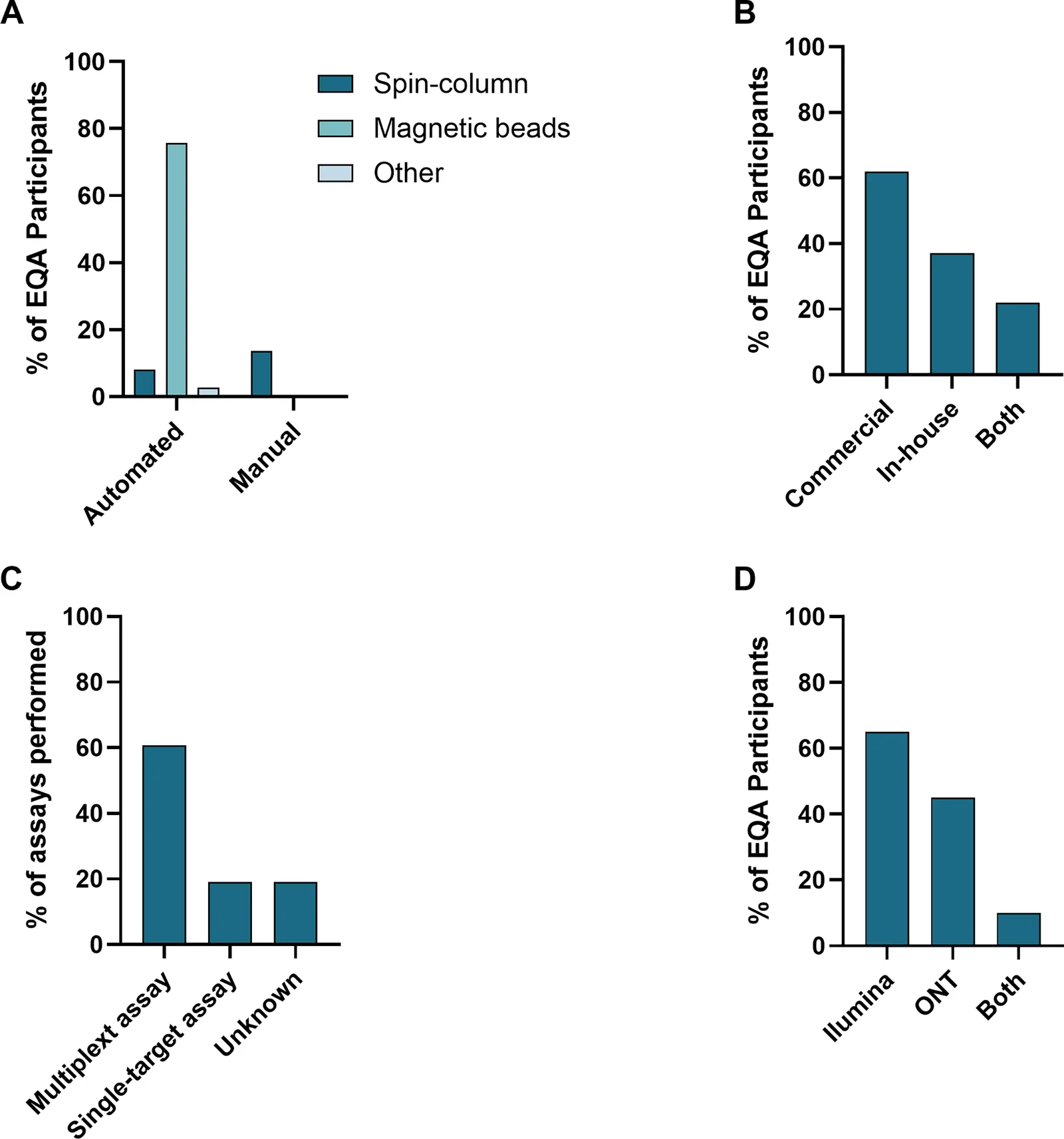
(**A**) Extraction methods used, (**B**) molecular assay types used, (**C**) percentage of single-target or multiplex assays used, and (**D**) NGS platforms used.

Sample extraction was mainly performed in an automated format (32/37; 86.5%). Only five laboratories extracted RNA manually (5/37; 13.5%). For automated extraction, magnetic bead-based approaches were mostly used (28/32; 87.5%) (Fig. 2A).

Commercial methods (23/37, 62%) were used more often than in-house assays (14/37, 37%), with eight laboratories (8/37; 22%) performing both (Fig. 2B and Table S2).

**Fig 3:**
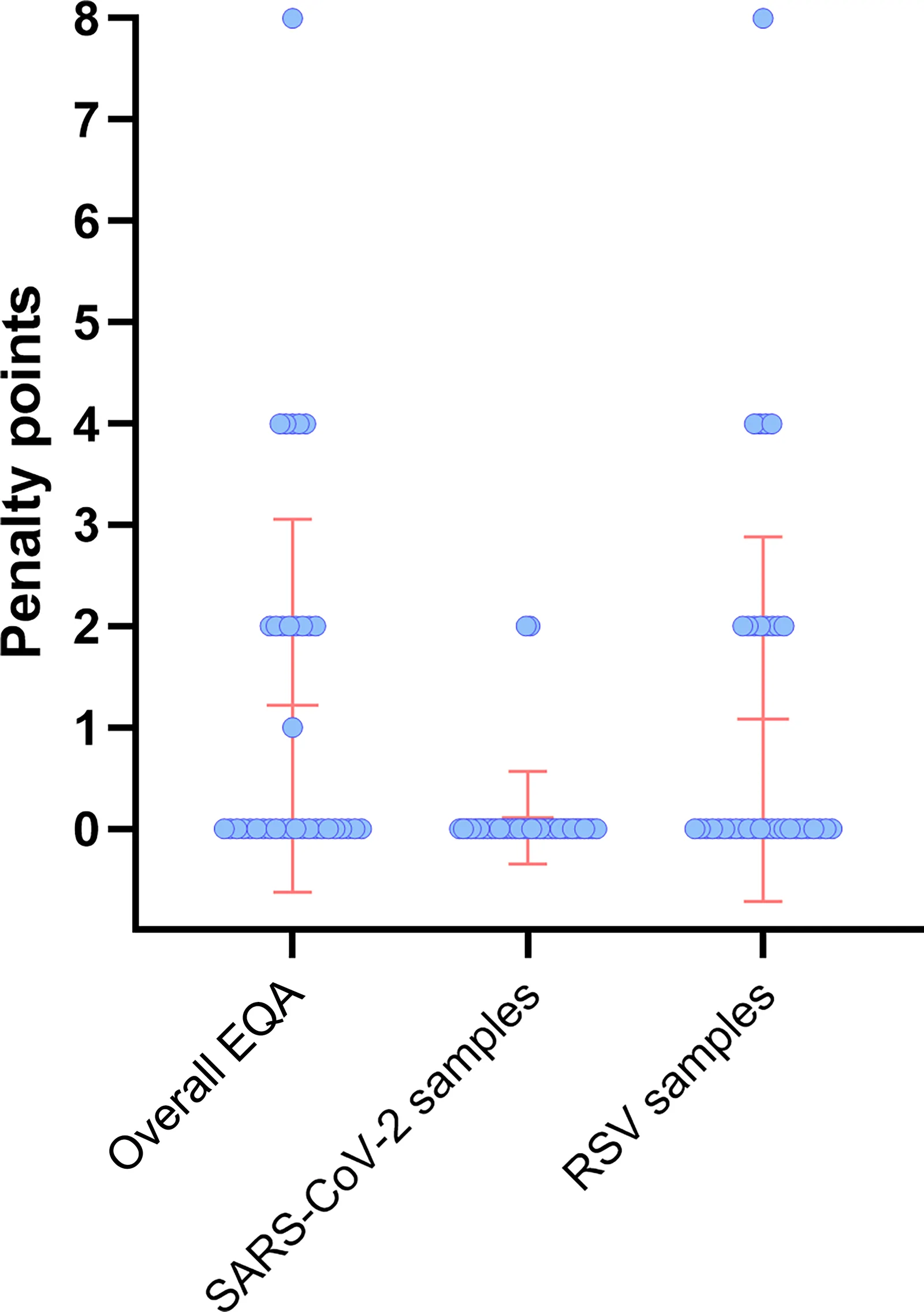
Total penalty points of all participating laboratories. Evaluation of the entire EQA, as well as penalty points for molecular detection of SARS-CoV-2-containing and RSV-containing samples. Correctly reported samples: 0 points; inconclusive results: 1 point, incorrect results: 2 points.

Sixty-two percent (23/37) of the participating laboratories identified all EQA samples correctly (0 penalty points; for explanation, please see Materials and Methods) (Fig. 3). Eight laboratories (22%) reported one false-negative result: one laboratory identified the SARS-CoV-2 B.1.17 sample (sample ID 1014) as negative, and seven others identified one RSV-containing sample as negative (mainly the low-concentration samples). One laboratory misidentified the RSV B.D.E.1 sample (sample ID 1041) as SARS-CoV-2-positive (Fig. 3A). This, however, was a technical form-filling error, given that both assays resulted in Ct values for the RSV-positive samples. One laboratory did not detect any of the RSV samples and reported them as negative despite using a commercial kit for multiple respiratory pathogens. The average score (see evaluation of results in Materials and Methods) for all participating laboratories was: 1 (range: 0–8; median: 0), where 0 means no penalty points (Fig. 3).

**Fig 4:**
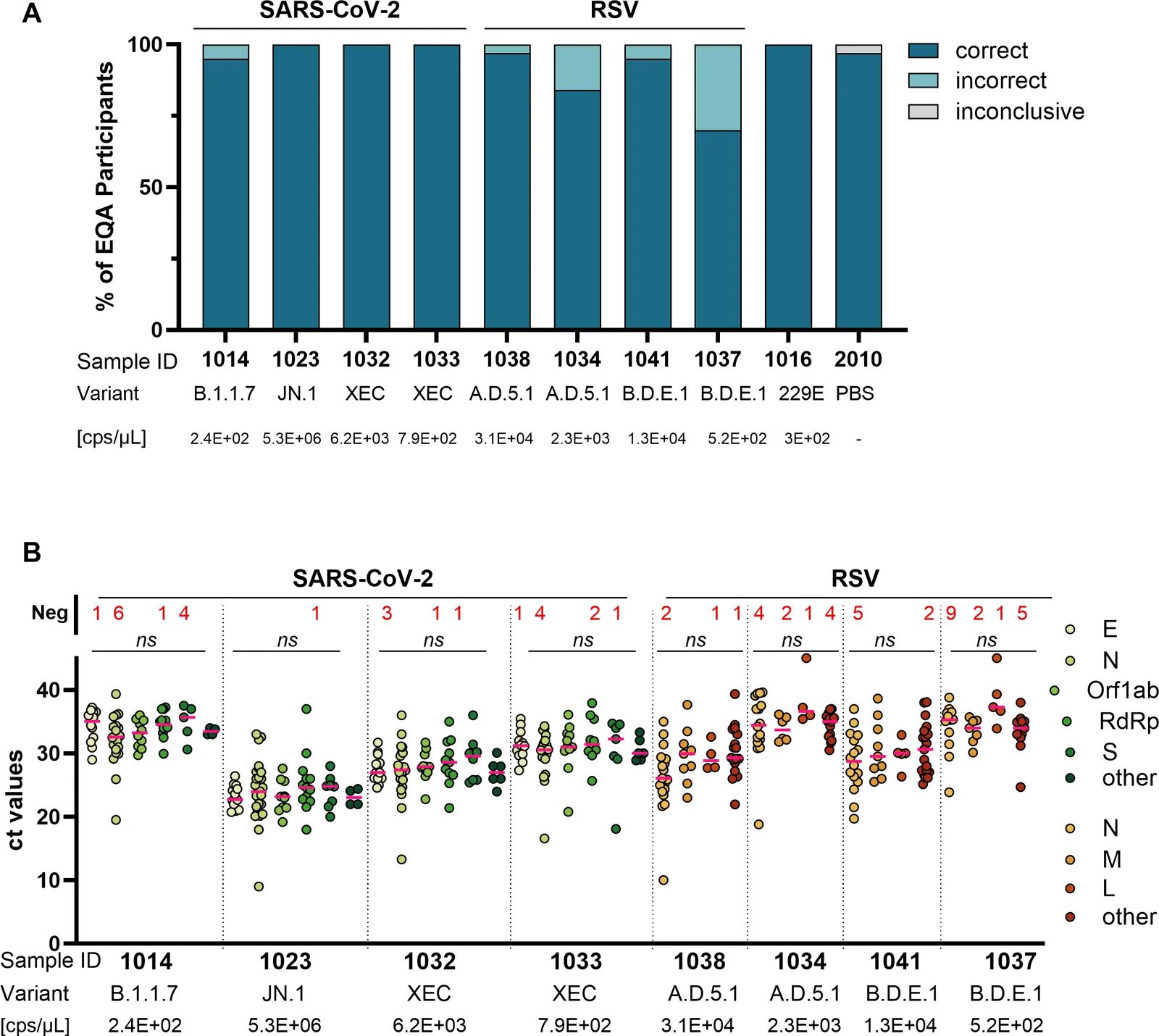
(**A**) Molecular detection of SARS-CoV-2 and RSV in the 2025 European wet-lab EQA. (**B**) Scatter dot plot of Ct values obtained for SARS-CoV-2- and RSV-containing samples by the amplification target genes used. Numbers in red above the plot indicate the number of negative results for the related assays. Pink horizontal line represents the median. Ns, not significant as, calculated using one-way ANOVA in GraphPad Prism (version 10.0). Other targets reported by participants were mostly unknown (due to commercial assays). Data for RSV F target are not shown (*n* = 1).

SARS-CoV-2 was detected by all the laboratories in three samples, including the XEC low-concentration sample. SARS-CoV-2 B.1.1.7 sample (sample ID 1014, 2.4E + 02 cps/µL), with the lowest SARS-CoV-2 concentration within this EQA panel, was detected by 35 out of 37 laboratories (95%). For the high-concentration RSV samples, RSV-A was detected by all other laboratories (36/37; 97%), while RSV-B was detected by 35/37 laboratories (95%). For the low-concentration RSV samples, five laboratories failed to detect both RSV-A and RSV-B, two laboratories missed RSV-A only, and six missed RSV-B only. These laboratories used a variety of commercial and in-house assays. Overall sensitivity (correctly determined positive samples) was 84% for RSV-A and 70% for RSV-B in the low-concentration samples. The negative and the HCoV-229E control samples were found negative for both RSV and SARS-CoV-2, indicating high specificity of the assays. One laboratory reported “inconclusive” for the PBS sample due to the lack of detection of human material but clarified that it represented a negative result (Fig. 4A).

The target genes used with obtained Ct values are shown in Fig. 4B. The main target genes for SARS-CoV-2 detection were E, N, ORF1ab, RdRp, and S. Other target genes were also used but not specified further. Regarding the detection of RSV, assays targeted N, M, and L genes, as well as other unspecified target genes. One laboratory also performed an F gene targeted assay, which was positive for the two high-concentration RSV-containing samples (data not shown). No significant difference in Ct values between the different assays and target genes was observed (Fig. 4B).

### RSV group determination

**Fig 5:**
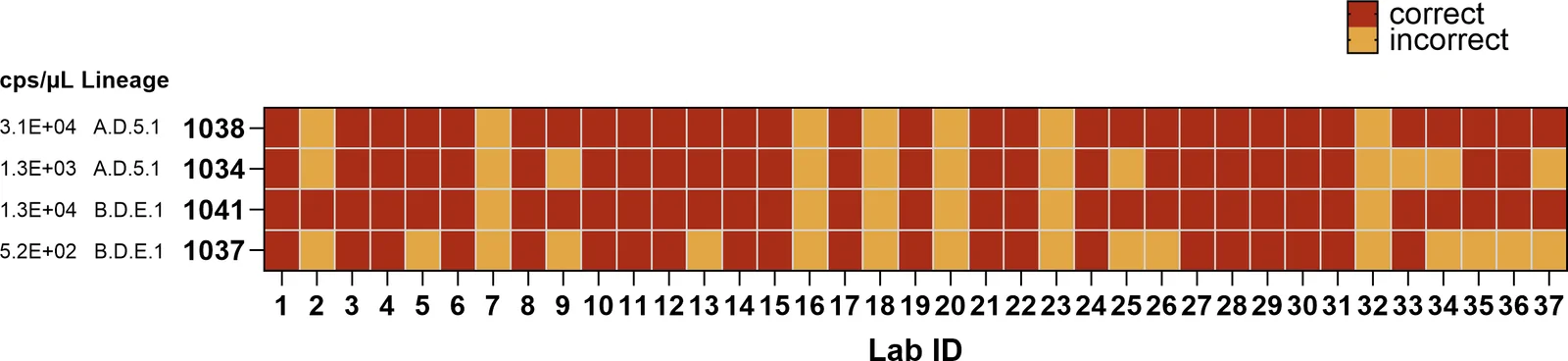
Group determination of the RSV-containing EQA samples by laboratory.

Group determination of RSV was a mandatory task. Twenty of 37 laboratories (54%) identified all RSV-containing samples correctly as group A or B. Six laboratories (6/37; 16%) correctly determined the group in three of the four RSV-containing samples, and four laboratories (4/37; 11%) correctly identified two of the four samples. Six laboratories (6/37; 16%) could not determine the RSV group in any of the samples (Fig. 5), mainly because they used commercial assays that did not differentiate between RSV groups.

### Sequencing of SARS-CoV-2 and RSV samples

A total of 20 laboratories participated in the optional task of performing NGS to determine SARS-CoV-2 variant or RSV lineage. One of these 20 also performed SARS-CoV-2 variant determination using a molecular method (SARS-CoV-2 variant PCR). In terms of platform, 13 laboratories (65%) used Illumina, and nine (45%) used Oxford Nanopore, including two laboratories (10%) that used both (Fig. 2D). One of these laboratories could not sequence any of the samples successfully. The other laboratory correctly identified two of the SARS-CoV-2-containing samples using Oxford Nanopore technology and two of the RSV-containing samples using Illumina sequencing. Different library preparation kits were used, including the Midnight RT PCR and Rapid Barcoding Kit 96 V14, Illumina Microbial Amplicon Prep, Native or Rapid Barcoding Expansion Kit 96, COVIDSeq Assay, Illumina DNA Prep, NEBNext Ultra DNA Library Prep Kit, Nextera XT DNA Library Preparation Kit, and the NEBNext ARTIC SARS-CoV-2 Library Prep Kit. A variety of sequencing analysis approaches and combinations were used, including in-house pipelines, commercial software, and online tools: BaseSpace, EPI2ME ARTIC SARS-CoV-2, EMC-Viroscience/SARS-CoV-2_submission, EIT Pathogena, DRAGEN, genepii/seqmet, INSaFLU, Nextclade, Nextstrain, NEXT-RSV-PIPE, Pangolin, poreCov, Ridom SeqSphere+, sarsseq, and ViroConstrictor. All laboratories performed amplicon sequencing, and one laboratory also performed a hybrid capture approach.

**Fig 6:**
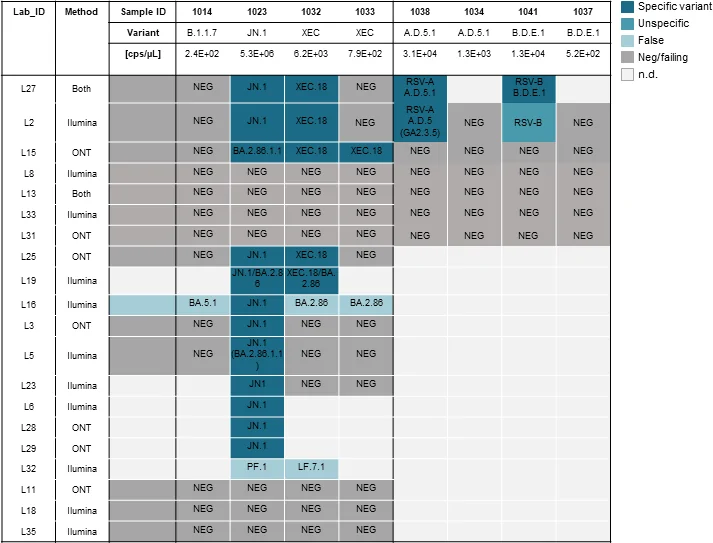
Overview of variant typing by NGS for SARS-CoV-2- and RSV-containing samples. NEG, negative result/unable to sequence/unassigned/not interpretable/not determined/not typable/low quality/not processable/not available.

Thirteen of 20 laboratories (65%) performing SARS-CoV-2 NGS succeeded in generating sequences (Fig. 6). Twelve of these correctly identified the JN.1 variant (sample ID 1023), the SARS-CoV-2-containing sample with the highest concentration. The remaining laboratory generated a sequence for this sample, which was, however, incorrectly reported as PF.1. Sample 1032 (XEC, high concentration) was correctly identified by five laboratories (25%), and one laboratory additionally correctly identified the low-concentration sample 1033 (XEC, low concentration). The sample containing B.1.1.7 (sample ID 1014) was not identified correctly by any of the laboratories, with most unable to generate a sequence. One laboratory misidentified this sample as BA.5.1.

Seven laboratories attempted NGS targeting RSV, and only two of them (29%) succeeded in generating sequences for further analysis. These laboratories identified the lineage of two RSV-containing samples with the higher concentration correctly. RSV-containing samples with a lower concentration were not identified by any laboratory (Fig. 6), either because no sequence could be generated or because no attempts were made after the samples were considered negative following molecular detection.

## DISCUSSION

We herein report on the 2025 European EQA for molecular detection of SARS-CoV-2, which also included, for the first time, RSV molecular detection and group determination. Participation rates and represented countries were comparable with previous EQAs that focused exclusively on SARS-CoV-2 (3).

In line with the previous EQAs, the molecular detection of SARS-CoV-2 in Europe has been steadily improving and reached 95% (35/37 laboratories) correctly identifying all samples in the current EQA (1–3). Issues were only seen with the low-concentration sample (2E + 02 cps/μL), although an improvement was observed compared to previous EQA where detection rates of those samples were around 90% (3).

This EQA brought an interesting conclusion that the molecular methods used by European expert laboratories are highly specific. No laboratory reported a false-positive result for the control samples. A single laboratory clarified that a reported inconclusive result for the PBS samples signifies lack of detection of human material, which is how their system was validated and designed. Such high specificity is reassuring, especially given that this EQA included three respiratory viruses: SARS-CoV-2, RSV, and HCoV-229E as one of the control samples.

In this EQA, 65% (24/37) of participating laboratories identified all RSV-containing samples correctly. Furthermore, 54% correctly identified the RSV groups for these samples. The main reasons for failure were no detection of RSV at all or lack of group determination within the assay used.

The optional task of sequencing was performed by 20 and seven laboratories for SARS-CoV-2 and RSV, respectively. While 65% (13/20) of laboratories successfully generated sequences for the SARS-CoV-2-containing samples, this was only the case for 29% (2/7) of the laboratories for the RSV-containing samples. A possible reason could be the performance of SARS-CoV-2 NGS using kits, while RSV sequencing is newer and generally relies on in-house assays. One limitation of this study was that the panel was designed with molecular detection in mind and also included low-concentration samples. These specimens evaluated sequencing capacities under more challenging conditions and, as expected, posed a challenge for the laboratories that opted to participate in the voluntary sequencing exercise. Only the higher-concentration samples could be sequenced for both SARS-CoV-2 and RSV samples. In the future, a panel with high-concentration samples could be designed specifically to evaluate sequencing capacities. Further issues to consider are the effects of BPL inactivation and lyophilization on sample quality. BPL treatment is widely used for viral inactivation but can also have a negative impact on viral samples, including aggregation of viral particles (10). Also, lyophilization could contribute to fragmentation or degradation of viral nucleic acids, thereby affecting downstream molecular analyses. Furthermore, transport of the samples, storage in the recipient laboratory, and sample processing may influence sequencing quality, especially when samples are processed with routine molecular detection in mind. During pre-testing of the EQA panel, we identified an inhibiting effect on the EQA samples, especially those with low viral load, presumably due to the sample preparation process. EQA sample generation needs to be adapted for future EQAs, especially when focusing on sequencing.

Dry-lab bioinformatic EQAs are organized simultaneously by the AURORAE consortium and ECDC (11). Many laboratories possibly opted not to participate in the optional sequencing exercise but rather focus on participating in the in-depth bioinformatics EQAs. A future synergy between those EQAs, e.g., by sharing samples in both, could help highlight where the gap lies. For SARS-CoV-2, given the excellent results and improvement trends from previous EQAs (1–3), continued surveillance of the ability to detect new variants, as well as more challenging tasks like sequencing, would be recommended.

With future EQAs for respiratory viruses in mind, more focus should fall on RSV detection, group, and lineage determination. Whether there is a clinical difference, particularly in terms of severity, between RSV-A and RSV-B remains unresolved. However, RSV-B appears to have a greater propensity for nirsevimab escape. Vaccines protect against severe forms of infection caused by both subtypes, including monovalent vaccines (which contain only the F protein of RSV-A). Nevertheless, emerging data suggest that antibody levels against RSV-B are lower, and subgroup-specific efficacy against RSV-B may decline more rapidly than against RSV-A with monovalent vaccines (12–14). In this context, A/B typing remains important for determining which viruses are circulating during a given season; it is more accessible than sequencing and can be performed on a larger number of samples. Sequencing also remains essential for defining lineages and identifying mutations that allow the virus to evade monoclonal antibodies. EQAs for RSV were previously performed by others, including WHO (15–19). The data from Quality Control for Molecular Diagnostics (QCMD) on RSV EQAs conducted between 2020 and 2023 in the WHO European region demonstrated high-quality diagnostic RSV detection and group identification (18). The authors, however, also highlighted the importance of continued quality assessments of RSV in the setting of multiple diagnostic assays for respiratory viruses in the aftermath of the COVID-19 pandemic. Furthermore, with the introduction of vaccines and new therapeutics for RSV, possible viral changes could hinder the ability of currently used diagnostic tests for proper viral identification (18). Evaluation of RSV sequencing performance of French laboratories found that sequence analysis accuracy varied for RSV-containing samples, with a wide variety of pipelines being used (19). In the study of Moisan et al., 10 of 13 (77%) and 11 of 12 (92%) of the laboratories correctly identified the G gene duplication for RSV-A and RSV-B strains, respectively, while 23% (three out of 13) of the laboratories made critical errors in the F protein gene with regard to SNPs (single-nucleotide polymorphism) (19). These data demonstrate the importance of organizing RSV molecular detection, sequencing, and sequence analysis-focused EQAs in the future. Additionally, European laboratories need to scale up their RSV sequencing capacity.

In general, continuous assessment of workflows and diagnostics for respiratory viruses is of high importance to ensure high-quality diagnostics, sustainable public health surveillance, and timely threat identification.

## SUPPLEMENTAL MATERIAL

Supplemental tables jcm.00258-26-s0001.docx Tables S1 to S2.

ASM does not own the copyrights to Supplemental Material that may be linked to, or accessed through, an article. The authors have granted ASM a non-exclusive, world-wide license to publish the Supplemental Material files. Please contact the corresponding author directly for reuse.
