# Supplementary material for: SARS-CoV-2 and RSV external quality assessment on molecular detection and variant typing in European expert laboratories in 2025
Source: J Clin Microbiol. 2026 Sep 17;64(10):e00258-26. doi: 10.1128/jcm.00258-26 (PMC13712985; doi:10.1128/jcm.00258-26)
Supplement: Supplementary file 1 [file jcm.00258-26-s0001.docx]

**Supplement Table 1. Molecular detection and sequencing of SARS-CoV-2 and RSV containing samples during panel pre-testing.**

|  |  | |  | | | **Ct vlaues^a^** | | |  |
| --- | --- | --- | --- | --- | --- | --- | --- | --- | --- |
| **Sample ID** | | **Virus** | | **Lineage** | | **Charité** | **RIVM** | **IP** |  |
| 1014 | | SARS-CoV-2 | | B.1.1.7 | | 35.48 | 32.23 | 35.64 |  |
| 1023 | | SARS-CoV-2 | | JN.1 | | 25.08 | 20.93 | 22.48 |  |
| 1032 | | SARS-COV-2 | | XEC | | 27.95 | 23.64 | 26.61 |  |
| 1033 | | SARS-CoV-2 | | XEC | | 31.2 | 27.91 | 30.92 |  |
| 1038 | | RSV | | A 2023-26978 (A.D.5.1) | | 27.98 | 22.03 | 24.73 |  |
| 1034 | | RSV | | A 2023-26978 (A.D.5.1) | | 34.94 | 29.76 | 34.64 |  |
| 1041 | | RSV | | B 2023-28159 (B.D.E.1) | | 30.47 | 23.71 | 23.52 |  |
| 1037 | | RSV | | B 2023-28159 (B.D.E.1) | | 35.17 | 31.23 | 33.46 |  |
| Charité | | Kit Library preparation | | | Kapa RNA Hyper Prep Kit, Roche | | | |  |
|  |  | Sequencing device | | | MiniSeq sequencing platform, Oxford Nanopore technologies | | | |  |
| RIVM | | Kit Library preparation | | | SQK-NBD114.96, Oxford Nanopore technologies | | | |  |
|  |  | Sequencing device | | | MinION sequencing platform, Oxford Nanopore technologies | | | |  |
| IP | | Kit Library preparation | | | NextSeq 500/550 High Output Kit v2.5, Illumina | | | |  |
|  |  | Sequencing device | | | NextSeq 500 sequencing platform, Illumina | | | |  |

^a^ Charité: SARS-CoV-2 in-house assay targeting E gene, RSVA/B commercial kit, Altona diagnostics. RIVM: SARS-CoV-2 in-house E gene assay, RSV A/B in-hoouse N gene assay. IP: SARS-CoV-2 CoVFlo2

**Supplement Table 2. Molecular assays used in the 2025 European SARS-CoV-2 and RSV EQA**

| **Assay** | **Type** | **Targets** | **Laboratories with all correct results. n** | **Comments on errors** |
| --- | --- | --- | --- | --- |
| CDC 2019-Novel Coronavirus (2019-nCoV) Real-Time RT-PCR Diagnostic Panel | multiplex | N (SARS-CoV-2) | 1/1 |  |
| CDC Influenza SARS-CoV-2 (Flu SC2) Multiplex Assay | multiplex | N (SARS-CoV-2). M (RSV). other non-SARS-CoV-2/RSV | 5/6 | N (SARS-CoV-2) No Ct 1014. 1016 |
| In-house Brittain-Long et al. ([https://doi.org/10.1016/j.jcv.2007.10.029](https://mcas-proxyweb.mcas.ms/certificate-checker?login=false&originalUrl=https%3A%2F%2Fdoi.org.mcas.ms%2F10.1016%2Fj.jcv.2007.10.029%3FMcasTsid%3D20892&McasCSRF=ca4b6eb5e1a7297c254a5bc23a665bcc0cea95f866195d00ce0016925644a0d4)) | multiplex | M (RSV) | 2/2 |  |
| In-house Corman et al. ([https://doi.org/10.2807/1560-7917.ES.2020.25.3.2000045](https://mcas-proxyweb.mcas.ms/certificate-checker?login=false&originalUrl=https%3A%2F%2Fdoi.org.mcas.ms%2F10.2807%2F1560-7917.ES.2020.25.3.2000045%3FMcasTsid%3D20892&McasCSRF=ca4b6eb5e1a7297c254a5bc23a665bcc0cea95f866195d00ce0016925644a0d4)) with variations | single | E (SARS-CoV-2). RdRp (SARS-CoV-2) | 5/5 |  |
| In-house Corman et al. ([https://doi.org/10.2807/ese.17.39.20285-en](https://mcas-proxyweb.mcas.ms/certificate-checker?login=false&originalUrl=https%3A%2F%2Fdoi.org.mcas.ms%2F10.2807%2Fese.17.39.20285-en%3FMcasTsid%3D20892&McasCSRF=ca4b6eb5e1a7297c254a5bc23a665bcc0cea95f866195d00ce0016925644a0d4)) | multiplex | ORF1ab (SARS-CoV-2) | 0/1 | ORF1ab (SARS-CoV-2) false negative 1014 |
| In-house Esposito et al. ([https://doi.org/10.1371/journal.pone.0129369](https://mcas-proxyweb.mcas.ms/certificate-checker?login=false&originalUrl=https%3A%2F%2Fdoi.org.mcas.ms%2F10.1371%2Fjournal.pone.0129369%3FMcasTsid%3D20892&McasCSRF=ca4b6eb5e1a7297c254a5bc23a665bcc0cea95f866195d00ce0016925644a0d4)) modified | multiplex | N (RSV) | 0/1 | N (RSV) false negative 1034. 1037. 1041 |
| In-house Hibino et al. ([https://doi.org/10.1371/journal.pone.0192085](https://mcas-proxyweb.mcas.ms/certificate-checker?login=false&originalUrl=https%3A%2F%2Fdoi.org.mcas.ms%2F10.1371%2Fjournal.pone.0192085%3FMcasTsid%3D20892&McasCSRF=ca4b6eb5e1a7297c254a5bc23a665bcc0cea95f866195d00ce0016925644a0d4)) | single | F (RSV) | 0/1 | F (RSV) false negative 1034. 1037 |
| In-house Hoek et al. ([https://doi.org/10.3109/00365548.2012.708942](https://mcas-proxyweb.mcas.ms/certificate-checker?login=false&originalUrl=https%3A%2F%2Fdoi.org.mcas.ms%2F10.3109%2F00365548.2012.708942%3FMcasTsid%3D20892&McasCSRF=ca4b6eb5e1a7297c254a5bc23a665bcc0cea95f866195d00ce0016925644a0d4)) | single | N (RSV) | 1/1 |  |
| In-house Hombrouck et al. ([https://doi.org/10.1007/s10096-011-1398-4](https://mcas-proxyweb.mcas.ms/certificate-checker?login=false&originalUrl=https%3A%2F%2Fdoi.org.mcas.ms%2F10.1007%2Fs10096-011-1398-4%3FMcasTsid%3D20892&McasCSRF=ca4b6eb5e1a7297c254a5bc23a665bcc0cea95f866195d00ce0016925644a0d4)) | single | N (RSV) | 1/1 |  |
| In-house Hu et al. ([https://doi.org/10.1128/jcm.41.1.149-154.2003](https://mcas-proxyweb.mcas.ms/certificate-checker?login=false&originalUrl=https%3A%2F%2Fdoi.org.mcas.ms%2F10.1128%2Fjcm.41.1.149-154.2003%3FMcasTsid%3D20892&McasCSRF=ca4b6eb5e1a7297c254a5bc23a665bcc0cea95f866195d00ce0016925644a0d4)) | single | N (RSV) | 0/1 | N (RSV) no Ct 1037. 1041 |
| In-house Kodani et al. (https://doi.org/10.1128/jcm.02270-10) | multiplex | M (RSV) | 0/1 | M (RSV) false negative 1034. 1037 |
| In-house Mackenzie et al. ([https://doi.org/10.1038/s41598-019-54059-4](https://mcas-proxyweb.mcas.ms/certificate-checker?login=false&originalUrl=https%3A%2F%2Fdoi.org.mcas.ms%2F10.1038%2Fs41598-019-54059-4%3FMcasTsid%3D20892&McasCSRF=ca4b6eb5e1a7297c254a5bc23a665bcc0cea95f866195d00ce0016925644a0d4)) | single | N (RSV) | 1/1 |  |
| In-house Raymenants et al. ([https://doi.org/10.1038/s41467-023-36986-z](https://mcas-proxyweb.mcas.ms/certificate-checker?login=false&originalUrl=https%3A%2F%2Fdoi.org.mcas.ms%2F10.1038%2Fs41467-023-36986-z%3FMcasTsid%3D20892&McasCSRF=ca4b6eb5e1a7297c254a5bc23a665bcc0cea95f866195d00ce0016925644a0d4)) | multiplex | L (RSV) | 1/1 |  |
| In-house Tiveljung-Lindell et al. ([https://doi.org/10.1002/jmv.21368](https://mcas-proxyweb.mcas.ms/certificate-checker?login=false&originalUrl=https%3A%2F%2Fdoi.org.mcas.ms%2F10.1002%2Fjmv.21368%3FMcasTsid%3D20892&McasCSRF=ca4b6eb5e1a7297c254a5bc23a665bcc0cea95f866195d00ce0016925644a0d4)) | multiplex | other non SARS-CoV-2 / RSV | 1/1 |  |
| In-house Tiveljung-Lindell et al. ([https://doi.org/10.1002/jmv.21368](https://mcas-proxyweb.mcas.ms/certificate-checker?login=false&originalUrl=https%3A%2F%2Fdoi.org.mcas.ms%2F10.1002%2Fjmv.21368%3FMcasTsid%3D20892&McasCSRF=ca4b6eb5e1a7297c254a5bc23a665bcc0cea95f866195d00ce0016925644a0d4)) | multiplex | L (RSV) | 1/1 |  |
| In-house Wang et al. ([https://doi.org/10.1016/j.jviromet.2019.113676](https://mcas-proxyweb.mcas.ms/certificate-checker?login=false&originalUrl=https%3A%2F%2Fdoi.org.mcas.ms%2F10.1016%2Fj.jviromet.2019.113676%3FMcasTsid%3D20892&McasCSRF=ca4b6eb5e1a7297c254a5bc23a665bcc0cea95f866195d00ce0016925644a0d4)) | multiplex | N (RSV) | 0/1 | N (RSV) false negative 1037 |
| In-house WHO 2019-nCoV ([https://www.who.int/publications/i/item/10665-331501](https://mcas-proxyweb.mcas.ms/certificate-checker?login=false&originalUrl=https%3A%2F%2Fwww.who.int.mcas.ms%2Fpublications%2Fi%2Fitem%2F10665-331501%3FMcasTsid%3D20892&McasCSRF=ca4b6eb5e1a7297c254a5bc23a665bcc0cea95f866195d00ce0016925644a0d4)) | single | RdRp (SARS-CoV-2) | 1/1 |  |
| In-house Williams et al. ([https://doi.org/10.1111/irv.13073](https://mcas-proxyweb.mcas.ms/certificate-checker?login=false&originalUrl=https%3A%2F%2Fdoi.org.mcas.ms%2F10.1111%2Firv.13073%3FMcasTsid%3D20892&McasCSRF=ca4b6eb5e1a7297c254a5bc23a665bcc0cea95f866195d00ce0016925644a0d4)) | single | L (RSV) | 1/1 |  |
| 1copy COVID-19/FluA/FluB/RSV qPCR Kit | multiplex | unknown SARS-CoV-2. unknown RSV | 1/1 |  |
| Alinity m Resp-4-Plex Kit | multiplex | N (SARS-CoV-2). RdRp (SARS-CoV-2).  unknown RSV. other non SARS-CoV-2 / RSV | 1/1 |  |
| Allplex Respiratory Panel 1A | multiplex | unknown RSV | 0/1 | RSV false negative 1037 |
| Allplex RV Master Assay | multiplex | N (SARS-CoV-2). RdRp (SARS-CoV-2). S (SARS-CoV-2). unknown RSV | 1/1 |  |
| Allplex SARS-CoV-2 Assay | multiplex | E (SARS-CoV-2). N (SARS-CoV-2). unknown SARS-CoV-2 | 2/2 |  |
| Allplex SARS-CoV-2 fast PCR Assay | multiplex | E (SARS-CoV-2). N (SARS-CoV-2). RdRp (SARS-CoV-2) | 1/1 |  |
| Allplex SARS-CoV-2/ FluA/ FluB/ RSV Assay | multiplex | N (SARS-CoV-2). RdRp (SARS-CoV-2). S (SARS-CoV-2). unknown RSV. other non SARS-CoV-2 / RSV | 0/4 | SARS-CoV-2 false negative 1014;  SARS-CoV-2 No Ct 1033;  RSV false negative/No Ct 1034. 1037.1041 |
| Argene RSV/hMPV r-gene | multiplex | N (RSV) | 0/1 | N (RSV) false negative 1034. 1037. 1041 |
| Argene SARS-CoV-2 Respi R-gene | multiplex | N (SARS-CoV-2). RdRp (SARS-CoV-2) | 1/1 |  |
| AusDiagnostics Respiratory Viruses 16-well | multiplex | ORF1ab (SARS-CoV-2).  unknown RSV | 2/2 |  |
| Cobas SARS-CoV-2 | multiplex | E (SARS-CoV-2). ORF1ab (SARS-CoV-2) | 1/1 |  |
| Diagnovital respiratory panel real time PCR kit | multiplex | unknown SARS-CoV-2.  unknown RSV | 1/1 |  |
| FTD Respiratory pathogens 21 | multiplex | other non SARS-CoV-2 / RSV | 1/1 |  |
| Genesig RSV | single | M (RSV) | 1/1 |  |
| Genesig RSV B | single | M (RSV) | 1/1 |  |
| LightMix Modular RSV | multiplex | M (RSV) | 1/1 |  |
| MutaPLEX® RespiraScreen 4 | multiplex | E (SARS-CoV-2). S (SARS-CoV-2).  unknown RSV | 1/1 |  |
| NxTAG Respiratory Pathogen Panel | multiplex | ORF1ab (SARS-CoV-2).  M (SARS-CoV-2). L (RSV) | 0/1 | L (RSV) false negative 1037 |
| R-Biopharm Respiratory viruses 16-well | multiplex | unknown SARS-CoV-2. unknown RSV. other non SARS-CoV-2 / RSV | 1/1 | RSV false negative 1037 |
| RealStar RSV RT-PCR kit 3.0 | multiplex | unknown RSV | 1/1 |  |
| Respiratory Tract RT-qPCR MX-24T Panel | multiplex | N (SARS-CoV-2). N (RSV) | 1/1 |  |
| Shanghai Kehua Bio-Engineering Diagnostic kit for SARS CoV-2 Nucleic Acid | multiplex | E (SARS-CoV-2). N (SARS-CoV-2). ORF1ab (SARS-CoV-2) | 1/1 |  |
| TaqPath COVID-19 CE-IVD RT-PCR Kit | multiplex | N (SARS-CoV-2). ORF1ab (SARS-CoV-2). S (SARS-CoV-2) | 1/1 |  |
| Viasure SARS-CoV-2 Triplex | multiplex | E (SARS-CoV-2). N (SARS-CoV-2). ORF1ab (SARS-CoV-2) | 1/1 |  |
| Xpert Xpress SARS CoV-2/Flu/RSV plus | multiplex | N (SARS-CoV-2). unknown RSV | 1/1 |  |
| XpertXpress Flu/RSV | multiplex | N (RSV) | 1/1 |  |
| Unknown In-house assay | Unknown/single/multiplex | Unknown/ varying targets | 12/17 | RSV false negative 1034. 1037. 1038. 1041 |
| Unknown commercial assay | Unknown/multiplex | Unknown/ varying targets | 6/12 | RSV false negative /No Ct 1034. 1037. 1038 |
